# From Adipose to Action: Reprogramming Stem Cells for Functional Neural Progenitors for Neural Regenerative Therapy

**DOI:** 10.3390/ijms26146599

**Published:** 2025-07-09

**Authors:** Junjie Peng, Zhu Zhang, Min Li, Ken Kin Lam Yung, King-ho Cheung

**Affiliations:** 1 Teaching and Research Division, School of Chinese Medicine, Hong Kong Baptist University, Kowloon Tong, Kowloon, Hong Kong SAR, China; 22482687@life.hkbu.edu.hk (J.P.); zhangzhu@hkbu.edu.hk (Z.Z.); limin@hkbu.edu.hk (M.L.); 2 Golden Meditech Centre for NeuroRegeneration Sciences, School of Chinese Medicine, Hong Kong Baptist University, Kowloon Tong, Kowloon, Hong Kong SAR, China; 3 Mr. & Mrs. Ko Chi Ming Centre for Parkinson’s Disease Research, School of Chinese Medicine, Hong Kong Baptist University, Kowloon Tong, Kowloon, Hong Kong SAR, China; 4 Department of Science and Environmental Studies, The Education University of Hong Kong, Tai Po, Hong Kong SAR, China

**Keywords:** adipose-derived stem cells, induced neural stem cell-like cells, Parkinson’s disease, regenerative medicine, stem cell therapy

## Abstract

Neural stem cells have shown great potential in the therapy of neurodegenerative diseases such as Parkinson’s disease (PD), because of their ability to differentiate into various types of neural cells and substitute for damaged neurons. Their clinical application is, however, impeded by limitations such as low survival rates following transplantation, low efficiency of differentiation, the potential for tumorigenesis, and the risk of immune rejection by the host. Adipose-derived stem cells (ADSCs) have become increasingly popular as an alternative tool in regenerative medicine due to their accessibility, multipotency, and low immunogenicity. The recent advance in inducing ADSCs into neural stem cell-like cells (iNSCs) opens up a new avenue for the treatment of PD by restoring dopaminergic neuron populations. Here, the biological characteristics, induction protocols, molecular mechanisms, and prospective applications of ADSCs in neural repair are summarized systematically. We also covered current technical challenges, such as differentiation protocol optimization and functional integration, and future perspectives, including biomaterial and gene editing applications to enhance ADSC-based therapies. With these challenges met, ADSCs hold excellent potential for advancing personalized and combination therapies for neurodegenerative diseases.

## 1. Introduction

Neurodegenerative diseases are a group of disorders characterized by the progressive degeneration of the structure and function of the nervous system. These conditions often lead to a decline in cognitive and motor functions, significantly impacting patients’ quality of life [1]. Among these disorders, Parkinson’s disease (PD) is the second most common neurodegenerative disorder. It is characterized by the gradual degeneration of dopaminergic neurons in the substantia nigra [2,3]. Existing treatments, such as dopamine replacement therapy and deep brain stimulation, ameliorate symptoms but do not stop disease progression or restore lost dopaminergic neurons [4]. Stem cell therapy is one of the promising strategies for replenishing dopaminergic neurons and repairing the disrupted neural circuit [5,6]. But the clinical use of conventional stem cell sources, including embryonic stem cells (ESCs) and induced pluripotent stem cells (iPSCs), is restricted due to ethical issues, tumorigenic potential, and immune rejection [7,8,9].

Adipose-derived stem cells (ADSCs) have been suggested as an alternative source of stem cells because of their accessibility, multipotency, and low immunogenicity [10,11]. In contrast to ESCs and iPSCs, ADSCs can be readily obtained from adipose tissue through less invasive procedures [12,13], making them an attractive candidate for autologous applications. Recently, autologous ADSC injections have gained popularity for treating central nervous system diseases. They can differentiate into neural stem cell-like cells (iNSCs) [14,15], suggesting a new strategy for the treatment of neurodegenerative disease, such as PD. Nonetheless, the molecular basis of ADSC transdifferentiation and their clinical use has not been well defined.

This review focuses on the biological properties, inducing methods, and molecular mechanisms of ADSCs differentiation into iNSCs. We also explore the therapeutic potential of ADSCs in neural regeneration. Additionally, we discuss existing technical challenges and future directions for optimizing ADSC-based therapies for PD. Most importantly, these challenges should be addressed as ADSCs have potential in progressing regenerative medicine and improving outcomes for neurodegenerative disease patients.

## 2. Characteristics of NSCs and ADSCs

Neural Stem Cells (NSCs) and ADSCs are both valuable tools in regenerative medicine because of their high capacity for self-renewal, multipotency for differentiation into different cell lineages, and ability to secrete paracrine factors [16]. However, they possess distinct biological features and therapeutic potentials. Below, we compare NSCs and ADSCs in terms of their sources, morphology, surface markers, and functional properties (Table 1).

### 2.1. Sources

#### 2.1.1. NSCs

The adult brain and embryonic tissues are the major sources of NSCs. In adults, NSCs are found in specific areas like the subventricular zone (SVZ) and the dentate gyrus of the hippocampal region [17,18]. NSCs can proliferate and differentiate into neurons, astrocytes, and oligodendrocytes after being isolated, which aids in neural plasticity and nervous system repair [16,19]. Microglia are not a typical differentiation product of NSCs because they originate from yolk sac-derived primitive myeloid progenitors [20]. Embryonic NSCs, on the other hand, originate from the neural tube during early development and exhibit a strong capacity of differentiation [18,21]. However, because of ethical issues and technical difficulties in their isolation and expansion, the use of embryonic NSCs is limited [22].

#### 2.1.2. ADSCs

ADSCs are mainly derived from the huge reserves of subcutaneous adipose [23,24]. They are typically harvested through less invasive techniques such as liposuction in humans or surgical extraction in experimental animals [25]. The harvested adipose tissue is enzymatically digested and processed to yield a cell suspension enriched with ADSCs. Notably, the typical yield of one gram of adipose tissue can be up to around 0.5 × 10^4^ to 2 × 10^5^ stem cells, significantly higher than the number of mesenchymal stem cells (MSCs; typically around 60–600 cells per 1 mL) obtained from bone marrow aspirate [26]. This abundance as well as the ease of isolation makes ADSCs an attractive choice for autologous transplantation and regenerative therapies [27,28].

### 2.2. Morphological Characteristics

#### 2.2.1. NSCs

**Figure 1:**
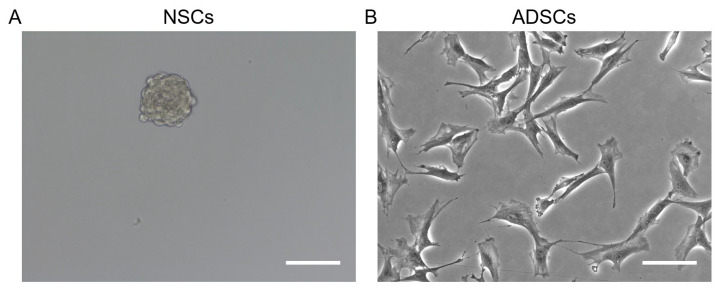
Morphological characteristics of NSCs (**A**) and ADSCs (**B**) from adult rats (scale bar: 50 µm).

In vitro, NSCs typically form neurospheres—free-floating clusters of cells—when cultured in specific media [29]. These neurospheres exhibit a multi-protrusion morphology (Figure 1A). Forming neurospheres is a hallmark feature of NSCs and reflects their ability of self-renewal and multipotency [30,31].

#### 2.2.2. ADSCs

ADSCs usually show a fibroblast-like, spindle-shaped morphology (Figure 1B) under standard culture conditions [32,33]. In contrast to the NSCs, ADSCs do not form neurospheres but they grow as an adherent monolayer instead (Figure 1B). While ADSCs share some morphological similarities with NSCs, their differentiation potential is primarily restricted to mesodermal lineages, like adipocytes, osteoblasts, chondrocytes, etc., unless specific induction protocols are applied [34,35].

### 2.3. Cell Surface Markers

#### 2.3.1. NSCs

NSCs express a range of specific surface markers. These markers include Nestin, Sox2, Musashi-1 and CD133, with each having its unique function [36]. Nestin, an intermediate filament protein, is an early marker of neural progenitor cells [37]. Sox2 is a transcription factor which plays a crucial role in maintaining the self-renewal and differentiation potential of NSCs [38]. Musashi-1 is an RNA-binding protein which regulates NSC proliferation and differentiation [39], while CD133 is a transmembrane protein that is associated with stem cell identity [40].

#### 2.3.2. ADSCs

ADSCs express mesenchymal stem cell markers such as CD29, CD90, and CD105 [41]. CD29, also known as integrin β1, mediates the adhesion of ADSCs to the extracellular matrix (such as collagen and laminin). It also regulates stem cell homing and differentiation [42]. CD90 (Thy-1) influences ADSC proliferation and differentiation through pathways such as Wnt/β-catenin [43], while CD105 (Endoglin) is involved in angiogenesis and tissue repair via the TGF-β/SMAD signaling pathway [44]. Importantly, ADSCs do not express hematopoietic makers like CD34 and CD45, ensuring their purity and suitability for clinical applications [45].

### 2.4. Functional Properties

#### 2.4.1. NSCs

NSCs possess distinctive capacity for self-renewal and differentiate into diverse neural cell types, including neurons, astrocytes, and oligodendrocytes [46]. Their multipotency arises from two division mechanisms: symmetric division produces two identical NSCs, whereas asymmetric division generates one NSC and one specialized cell [47]. These cells are essential for neural repair and regeneration processes, positioning them as promising therapeutic candidates for neurodegenerative diseases [48,49].

#### 2.4.2. ADSCs

ADSCs demonstrate exceptional multipotency, differentiating into multiple cell lineages such as osteoblasts, chondrocytes, adipocytes, hepatic lineage, and even neural-like cells under specific induction conditions [50,51]. Besides differentiation capabilities, ADSCs also secrete several neurotrophic factors, including the nerve growth factor (NGF), the brain-derived neurotrophic factor (BDNF), neurotrophin-3 (NT-3), the ciliary neurotrophic factor (CNTF), the glial cell-derived neurotrophic factor (GDNF), and artemin [41,52,53]. These bioactive molecules induce neural repair, minimize inflammation responses, and augment tissue regeneration [54]. Moreover, ADSCs release extracellular vesicles (EVs) that carry anti-inflammatory and neuroprotective effects, suggesting their potential application in cell-free therapy approaches [55,56].

**Table 1:** Comparison of NSCs and ADSCs.

Characteristics	NSCs	ADSCs	References
Sources	Embryonic NSCs (neural tube region); Adult NSCs (hippocampus, SVZ); ESCs; iPSCs	Adipose tissue (subcutaneous fat)	[17,49]
Morphological Characteristics	Form neurospheres in specific culture medium; Spindle-shaped or multi-protrusion morphology	Fibroblast-like, spindle-shaped morphology	[30,57]
Differentiation Potential	Neurons; Astrocytes; Oligodendrocytes	Adipocytes; Osteoblasts; Chondrocytes; Hepatic lineage; Neural cells	[34,58]
Surface Markers	Nestin; Sox2; CD133; Musashi-1	CD9, CD10, CD13, CD29, CD73, CD90, CD105, CD271; Do not express HSCs markers (CD31, CD45, CD11B)	[41,59,60]
Neurotrophic factors	NGF, BDNF, GDNF, IGF-1; TGF-β; IGF1	VEGF, EGF, HGF, IGF1, PGDF, FGF, TGF-β, BDNF, GDNF, NGF	[52,61,62,63,64]
Proliferation Capacity	Self-renewal through symmetric and asymmetric division	Self-renewal and long-term proliferation capacity in vitro	[18,56]
Immunogenicity	Allogeneic transplantation may trigger immune responses	Suitable for autologous transplantation	[65,66]
Special effect	-	Secreting cytokines and exosomes; EVs	[54,55,56,67,68]

## 3. Research Methods of ADSCs-to-iNSCs Induction Process

**Figure 2:**
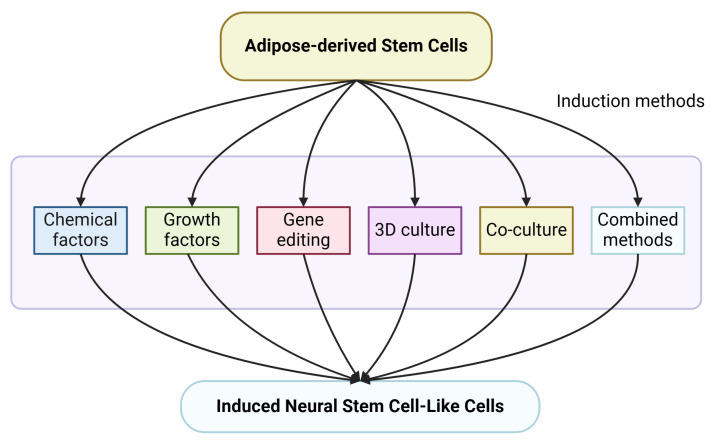
Induction processes of ADSCs-to-iNSCs, including chemicals, growth factors, gene editing technology, three-dimensional culture system, co-culture and combined induction method; the detailed protocols are contained in Table 2.

ADSCs have emerged as a promising source for iNSCs generation owing to their accessibility, multipotency, and lower immunogenicity [49,53]. Various induction methods were established for differentiation of ADSCs into iNSCs with specific advantages and limitations associated with each [11]. The most widely used induction strategies are described herein, such as chemical factors, growth factors, gene editing, 3D culture, co-culture, and combination approaches (Figure 2 and Table 2).

### 3.1. Chemical Induction

Chemical induction is achieved by using certain chemicals that stimulate signaling pathways to differentiate ADSCs into neural cells. Retinoic acid (RA), β-mercaptoethanol (BME), sertraline, valproic acid (VPA), butylated hydroxyanisole, forskolin, and L-carnitine (LC), etc., are some of the commonly used chemical inducers.

*Retinoic acid (RA):* RA, a metabolite of vitamin A, is a ligand for nuclear RA receptors (RARs) and an essential factor in neural development. It induces neural progenitors gene expression like Sox1 and Sox2, and suppresses mesodermal differentiation [69,70].

*β-mercaptoethanol (BME):* BME is a reducing agent that maintains the intracellular redox balance, supporting cell survival and differentiation [71]. BME pre-treatment and subsequent neural induction medium (NIM) have been shown to induce ADSCs into neural progenitor cells [72,73].

*Valproic acid (VPA):* VPA, an inhibitor of histone deacetylase, induces mature neuronal differentiation of ADSCs through the regulation of calcium signaling and nitric oxide pathways [74,75]. Furthermore, VPA improves the efficiency and specificity of induction by synergistically interacting with other chemical inducers [76].

*L-carnitine (LC):* LC, a derivative of amino acid [77], promotes neurogenic differentiation through the activation of Wnt/β-catenin and the protein kinase A (PKA) pathway [78].

*Other chemical inducers:* Sertraline is a synthetic chemical of the selective serotonin reuptake inhibitors (SSRIs) drug class and is used for the treatment of depression, anxiety, obsessive-compulsive disorder, and other psychosocial disorders [79]. It was found that sertraline promotes ASDCs’ proliferation and differentiation, whereas it inhibits the gliogenesis of ADSCs [80].

Chemical induction offers a cost-effective and straightforward approach to ASCDs’ differentiation into iNSCs. These chemicals activate the critical signaling pathways involved in neural development, making them accessible and easy-to-use reagents for researchers. The approach is, nonetheless, beset by drawbacks that include low specificity because the chemical may have off-target effects and needs careful optimization to achieve reproducible and efficient differentiation.

### 3.2. Growth Factors

Growth factors are essential for the activation of the in vivo microenvironment and to induce neural differentiation. The most significant growth factors are B27, basic fibroblast growth factor (bFGF), brain-derived neurotrophic factor (BDNF), epidermal growth factor (EGF) and human platelet lysate (HPL) [81,82].

The EGF and bFGF are commonly utilized in combination to induce neurosphere formation and maintain the self-renewal of neural progenitor cells [83]. In 2004, non-human primate adipose tissue stromal cells were successfully induced to develop into neurospheres in a B27, bFGF, BDNF and EGF-supplemented culture medium [84]. From 2014, Feng et al. set up a three-step induction protocol to differentiate very pure NSCs from human ADSCs through an activation of SOX1 by a conditioned medium with EGF and bFGF [81]. Later, ADSCs were induced to develop neurospheres with NSC-like properties through a neurobasal medium containing EGF and bFGF-2, and B27 [85,86,87,88]. More recently, homogenous cell populations of proliferating ADSCs cells have been induced to differentiate into iNSCs in culture media supplemented with EGF, bFGF, N2, and B27 [89]. ADSCs are differentiated to cells with a Schwann cell phenotype when cells are exposed to a combination of glial growth factors (GGF-2, bFGF, and PDGF) [90]. BDNF supports neuronal differentiation and survival through the activation of TrkB receptors [91]. Apart from these general growth factors, certain other factors, such as HPL and ghrelin, have also been utilized for differentiating ADSCs. Since HPL contains neurotrophic factors such as NTF3, BDGF, GDNF, and NGF, it is a potent inducer of neural differentiation [92].

While growth factors provide a very specific and effective means of inducing neural differentiation, their high cost and variability in stability, along with the need for stringent control over their concentration and timing, pose significant limitations to their large-scale application.

### 3.3. Gene Editing Technology

Gene editing techniques, for example, lentivirus or retrovirus transduction, enable targeted overexpression of neural-specific genes like *Sox2*, *OCT4*, and *KLF4* in ADSCs, which trigger them to differentiate into iNSCs. These processes are highly specific and long-lasting due to stable genetic modification, making them efficient tools to yield functional neural cells. In addition, forced expression of *Sox2* has been reported to directly induce the NSC phenotype in ADSCs [93], while multi-gene editing including *OCT4*, *KLF4*, *Sox2*, and *c-MYC* can reprogram ADSCs into iPSCs for subsequent neural differentiation [94]. However, gene editing is faced with challenges such as ethical concerns, technical complexity, and the risk of off-target effects or tumorigenicity, which need to be addressed carefully to ensure it offers safe and effective clinical translation.

### 3.4. Three-Dimensional (3D) Culture System

3D culture systems such as fibrin matrix and hydrogel scaffolds present a physiologically relevant environment mimicking the biochemical and mechanical properties of the central nervous system [95]. These systems enhance ADSC differentiation into iNSCs by promoting cell–cell interactions, neurosphere formation, and the expression of neural markers. For example, fibrin-based matrices induce stable ADSC differentiation into neural progenitor cells, while PEG-based hydrogels can induce spontaneous neural differentiation and enhance cell proliferation [96]. While 3D culture systems represent a more in vivo-like environment for neural differentiation, their complicated setup, scaffold characteristic variability, and requirement for precise optimization of mechanical and biochemical cues are challenges to standardization and scaling up in clinical applications.

### 3.5. Co-Culture Induction

Induction of co-culture allows ADSCs to differentiate into iNSCs through the utilization of direct or indirect interaction with other cell types, i.e., NSCs or olfactory ensheathing cells (OECs) [97]. Direct co-culture induces neural differentiation via physical cell–cell contact, whereas indirect co-culture uses soluble factors secreted from supporting cells to establish a neural-inductive microenvironment. For instance, co-culture of NSCs with ADSCs was found to improve neural marker expression and functional recovery [16], and OEC-conditioned medium is able to guide neural differentiation in the absence of direct contact [98]. While co-culture systems better mimic natural developmental signaling, they are limited by the availability of such specific cell types and are also problematic for standardization, thus rendered less feasible in large-scale applications [99].

### 3.6. Combined Induction

Combination methods integrate two or more induction approaches, including chemical reagents, growth factors, and gene editing, to differentiate ADSCs into iNSCs in an enhanced and targeted way. With combination strategies such as bFGF and EGF combined with chemicals or overexpression of neural-specific genes in a 3D culture environment, they realize the elevated expression of neural markers and functional characteristics via synergistic effects [81,100,101]. For instance, studies have indicated that the application of small molecules (e.g., SB431541 and noggin) together with growth factors (e.g., EGF and bFGF) improves the neural differentiation potential of ADSCs [102]. Although the combination approaches provide improved induction efficiency and functional integration, their greater complexity, higher cost, and requirement for precise optimization of multiple factors are barriers to clinical translation and scalability.

**Table 2:** Induction methods of ADSCs into iNSCs.

Induction Methods	Key Factors/Techniques	Advantages	Challenges	References
Chemical Factor	RA; BME; Forskolin; Sertraline; VPA; VPA + butylated hydroxyanisole + insulin + hydrocortisone; LC; BMP4	Cost-effective; Easy to implement	Limited specificity; Potential off-target effects	[50,70,72,73,74,75]
Growth Factor	BDNF; GDNF; EGF; bFGF; NGF; TGF-β; N2; B27; Ghrelin; FGF2	High specificity and efficacy	High cost and potential instability of growth factors	[81,85,86,87,88]
Gene Editing	Sox2; CGRP; OCT4; KLF4; SOX2; and c-MYC	High precision; Long-lasting effects	Ethical concerns, off-target effects; Technical complexity	[89,94,103]
3D Culture System	Fibrin matrix microenvironment; Hydrogel scaffold; PEG-Based 3D Matrix	Better mimics in vivo conditions	Complex setup; Potential variability	[95,96,104]
Co-Culture Induction	Direct contact co-culture; No-contact co-culture with ESCs; Chitosan co-culture Systems	Utilizes natural signaling mechanisms	Requires access to other cells	[16,99,105]
Combined Induction	Melatonin + CM; Indomethacin + Insulin + IBMX + PBM; Sox1 Activation + CM; bFGF + forskolin; BDNF + RA; SB431542 + noggin + LDN193289 + EGF + bFGF; 3D hydrogels + B27 + C1; Insoluble fibrin supported adhesion matrix + growth factors;	Maximizes induction efficiency/outcomes	Increased complexity and cost	[81,100,101,102,106,107,108,109]

In summary, compounds, growth factors, gene editing, 3D culture, co-culture, and various combinations can induce ADSC differentiation into iNSCs (Figure 2 and Table 2). In addition, there are still some problems in validating the method of inducing ADSC differentiation. There are currently few in vivo studies on the differentiation of ADSC into iNSCs, and most studies only focus on the expression of surface markers, so the stemness and differentiation functions after induction have not been fully verified. Hence, the best combination of factors remains to be determined.

## 4. Molecular Signal Pathways of ADSCs-to-iNSCs Induction Process

**Figure 3:**
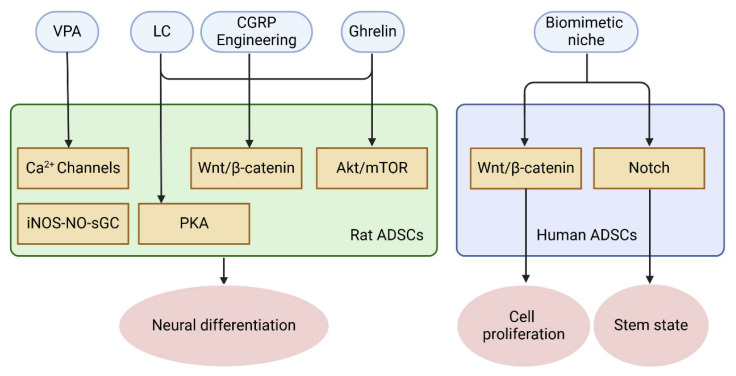
Molecular signal pathways of ADSCs-to-iNSCs induction process.

The differentiation of ADSCs into iNSCs is governed by a complex molecular signaling network [110]. Chapter 3 outlines various approaches to induce stem cell differentiation, while this chapter delves into the signaling pathways that mediate these effects. Induction methods trigger specific signaling pathways that guide stem cells to their target fate, highlighting the interaction between external cues and cellular responses. Notch, Wnt/β-catenin, and Akt/mTOR pathways, among many others, control the process of reprogramming by influencing gene expression and cellular activities [111,112]. The following is an overview of the major pathways in ADSC-to-iNSC differentiation and their roles in neural development (Figure 3 and Table 3).

### 4.1. Notch Signaling Pathway

The Notch pathway is needed for maintaining the undifferentiated phenotype of iNSCs through the inhibition of premature neuronal differentiation [109]. Activation occurs when Notch receptors bind to ligands from neighboring cells, triggering proteolytic cleavage and release of the Notch intracellular domain (NICD). The NICD translocates to the nucleus, influencing gene expression by interacting with transcription factors [113]. In human ADSCs, notch signaling along with fibrin-based niche elements control the fate of neural progenitor cells [113,114]. Notch signaling inhibition was found to facilitate neuronal differentiation, thereby indicating its function as a gatekeeper of neural commitment [115].

### 4.2. Wnt/β-Catenin Signaling Pathway

Wnt signaling is crucial for stem cell self-renewal and differentiation. In the canonical pathway, Wnt proteins bind to Frizzled receptors, stabilizing β-catenin, which then enters the nucleus to modulate gene expression [116]. The Wnt/β-catenin pathway plays context-dependent roles in ADSCs differentiation. In rat ADSCs, ghrelin-activated Wnt/β-catenin strongly induces neurogenic differentiation [92]. The pathway also works together with fibrin-based matrices to cause neural progenitor cell proliferation and differentiation [109,117]. In addition, Wnt/β-catenin signaling also synergizes with other pathways such as PKA, to drive neural differentiation [118,119].

### 4.3. Akt/mTOR Signaling Pathway

The Akt/mTOR pathway regulates cell survival, growth, and differentiation [120]. Stimulating this pathway in ADSCs with ghrelin activates biphasic regulation of Wnt/β-catenin and mTOR signaling, thereby inducing neural differentiation [78,92,94,109]. Its crosstalk with Wnt and Notch pathways underscores its integrative role in ADSC-to-iNSC reprogramming. The pharmacological regulation of Akt/mTOR has also been reported to enhance the effectiveness of ADSC-to-iNSC differentiation and therefore represents a therapeutic target opportunity [92].

### 4.4. Calcium Signaling and Redox Regulation

Calcium signaling and redox regulation are critical modulators of ADSC differentiation. VPA-treated ADSCs have functional N-type voltage-gated Ca^2+^ channels, which induce mature neuronal commitment [75]. In addition, the VPA-induced iNOS-NO-sGC axis is involved in neural differentiation induction [74]. These findings highlight the importance of calcium and redox signaling in ADSC reprogramming.

### 4.5. Multi-Pathway Crosstalk

ADSCs reprogramming into iNSCs entails the hierarchical coordination of multiple signaling pathways. LC, for instance, induces neurogenesis via the simultaneous activation of PKA and Wnt/β-catenin [78], whereas ghrelin possesses dual regulatory roles via Wnt/β-catenin and Akt/mTOR [92]. Such interactions highlight the intricacy of ADSC reprogramming and system-level appreciation of signaling networks.

These pathways do not operate in isolation; they form a dynamic network that integrates external signals and intrinsic cellular states. The interplay between Notch, Wnt/β-catenin, and Akt/mTOR pathways exemplifies the complexity of molecular mechanisms driving ADSC-to-iNSC differentiation. A systematic understanding of these interactions provides insights into potential therapeutic strategies for enhancing neural differentiation. Each pathway plays a critical role in regulating stem cell fate, with the Notch pathway maintaining the undifferentiated state, while Wnt/β-catenin and Akt/mTOR pathways facilitate neurogenic differentiation through various stimuli. Additionally, the interactions among these pathways emphasize the intricate crosstalk involved in ADSC reprogramming, enhancing our knowledge of the neural potential of ADSCs and opening up avenues for improving ADSC-derived iNSC differentiation.

**Table 3:** Molecular mechanisms of ADSCs-to-iNSCs induction process. hADSCs: human ADSCs; rADSCs: rat ADSCs.

Signaling Pathway	Source	Induction Methods	Description	References
Notch	hADSCs	Biomimetic niche	Maintains the undifferentiated state of iNSCs	[107]
Wnt/β-catenin	hADSCs	Biomimetic niche	Induces cell proliferation	[109]
	rADSCs	Ghrelin	Promotes neural differentiation	[92]
	rADSCs	LC	Promotes neural differentiation	[78]
	rADSCs	CGRP gene-editing	Promotes neural differentiation	[94]
Calcium (Ca^2+^) and ROS	rADSCs	VPA	Promotes neural differentiation	[75]
iNOS-NO-sGC	rADSCs	VPA	Promotes neural differentiation	[74]
Akt/mTOR	rADSCs	Ghrelin	Promotes neural differentiation	[92]
PKA	rADSCs	LC	Promotes neural differentiation	[78]

## 5. Application Prospects

To date, ADSCs and their induced iNSCs have shown great potential in the therapy of PD and other neurological disorders [121,122,123]. Their abundance, multipotency, and low immunogenicity render them an effective alternative to conventional stem cell sources. Here, the therapeutic prospectives of ADSCs and iNSCs for PD, their utility in drug and neurotoxicity assessment, and tissue engineering, as well as clinical translation challenges, will be elaborated (Table 4).

### 5.1. Therapeutic Potential in Parkinson’s Disease

ADSCs exhibited neuroprotection in PD preclinical models, such as the protection of dopaminergic neurons and inhibition of neuroinflammation [122,124]; especially, ADSC-EVs could inhibit the activation of microglia and protect neurons from apoptosis [125]. Also, ADSCs are alive, migrate, and become functionally integrated into damaged neural tissues upon transplantation, where they differentiate into tyrosine hydroxylase (TH)-positive dopaminergic neurons and ameliorate motor deficits [126,127]. In comparison to NSCs, ADSCs also have the advantages of being simply isolated from autologous adipose tissue, having low immunogenicity, and not requiring invasive brain surgery [128]. While NSCs have more robust neural differentiation capability, their use is hampered by ethical issues, risks of tumorigenicity, and poor survival of grafts [129].

In animal models, ADSCs have been induced to differentiate into dopaminergic neurons and restore motor dysfunction [123,130]. For example, ADSCs induced in a neurogenic differentiation medium and grafted into 6-hydroxydopamine (6-OHDA)-lesioned rats survived as dopaminergic neurons and restored the motor function of the animal [131]. Similarly, grafting of LMX1A-overexpressing ADSCs with adenoviral delivery of neurturin (NTN) and TH (Ad-NTN-TH) enhanced dopaminergic gene expression and conferred superior neuroprotection in MPTP-lesioned hemiparkinsonian rhesus monkeys [132]. These results demonstrate the potential of ADSCs as a treatment candidate for PD.

### 5.2. Drug and Neurotoxicity Assessment

Apart from direct cell therapy, ADSCs offer significant therapeutic potential for enhancing the efficacy of certain drugs. ADSCs loaded with palm oil ester-coated magnetic nanoparticles represent a promising strategy for targeted drug delivery to solid tumors, showing that ADSCs maintain high viability and motility while effectively carrying anti-tumor drugs [133]. Furthermore, ADSC-derived iNSCs also offer an invaluable tool for drug and neurotoxicity testing. The cells allow for in vitro screening of neuroprotective or regenerative medications, which diminishes the need for animal studies and avoids ethical restrictions [134]. For instance, iNSCs have been employed to model PD in vitro, which has enabled researchers to investigate disease mechanisms and screen for putative therapies in a controlled environment [135].

### 5.3. Neural Tissue Engineering

ADSC-derived iNSCs are also being investigated as precursor cells for neural tissue engineering. When used in combination with biomaterials like 3D-printed scaffolds, iNSCs facilitate nerve regeneration and increase axonal regrowth in injured tissues [95,100,136]. These strategies can reconstitute neural circuits and restore function in patients with PD and other neurological disorders.

Although ADSC-based therapies show promise, several issues must be addressed before they can be successfully implemented in clinical settings. These challenges include optimizing differentiation conditions to promote functional incorporation, resolving safety concerns related to tumorigenicity, and scaling up production for broader use. Additionally, long-term efficacy studies are essential to evaluate the effectiveness and safety of ADSC-derived iNSCs in human patients. In the next chapter, we will explore specific solutions to these challenges and outline future research directions.

**Table 4:** Therapeutic applications of ADSCs and iNSCs in neurological disorders.

Application	Key Findings	References
Dopaminergic neuron replacement	ADSCs differentiate into TH-positive neurons and improve motor deficits in PD models	[126,127]
Neuroprotection	ADSCs suppress neuroinflammation and preserve dopaminergic neurons	[123,125,137]
Drug assessment	ADSCs and iNSCs provide a platform for identifying neuroprotective compounds and drug delivery	[57,133]
Neural tissue engineering	iNSCs combined with biomaterials promote nerve regeneration and axonal regrowth	[136]

## 6. Research Challenges and Future Prospects

Although significant progress has been made in developing ADSC-induced iNSCs for PD treatment, some critical challenges have to be addressed before clinical implementation.

### 6.1. Technical Bottlenecks

The current induction protocols (Table 2) are highly heterogeneous, with differentiation efficiencies varying based on technique and donor variables. While chemical induction approaches using compounds like VPA and RA are relatively inexpensive and straightforward, they produce heterogeneous populations with partial neural commitment. Growth factor-based strategies have enhanced specificity but need precise optimization of time and dose to prevent overstimulation. Even more sophisticated techniques, including 3D cell culture and gene manipulation, more closely replicate physiology at the expense of increased complexity of standardization and scale.

Single-cell sequencing (scRNA-seq) studies have uncovered inherent ADSC heterogeneity as a significant limiting factor, with only 20–30% of cells attaining full neural reprogramming [103,138]. This heterogeneity is highly donor age- and harvest site-dependent [139,140,141,142]. Recent research has revealed the pseudo-temporal dynamic evolution characteristics of ADSCs during their differentiation into neuronal cells via scRNA-seq [143]. This approach facilitates the selection of specific neuronal cell types from ADSCs post-induction, effectively reducing heterogeneity.

### 6.2. Clinical Translation Challenges

The translation from preclinical studies to clinical applications has particular difficulties for ADSC-based therapies [144]. While phase II trials have moved forward with 1200 patients with the use of pluripotent stem cells [6], ADSC-derived neural precursors face unique difficulties. One of the key concerns is the stability of induced phenotypes because up to 40% of chemically derived “neural-like” cells can dedifferentiate back to their original state or exhibit stress-related artifacts [7,145]. Prolonged in vitro culture beyond passage 10 risks karyotypic abnormalities [146], and thus necessitate diligent monitoring of genomic integrity.

The stromal vascular fraction, which is obtained from adipose tissue, contains functionally heterogeneous subpopulations [147], and evidence suggests that the CD271^+^/CD146^+^ subsets have strong neural differentiation potential [139,148,149,150]. Such biological complexity highlights the need to standardize isolation and characterization protocols. In addition, the degree to which cell replacement and paracrine mechanisms underlie beneficial effects is unclear, which makes it more difficult to further optimize the dose and choose it for delivery strategy.

### 6.3. Future Directions

Several key priorities emerge for advancing ADSC-based neural therapies for PD. First and foremost is the requirement of protocol optimization. This involves the development of GMP complaint induction protocols with AI-backed quality control. Functional validation assays to enhance consistency and reliability of cell production are also important. Second, mechanistic studies founded on single-cell multi-omics approaches are required to identify molecular signatures for predicting successful neural differentiation and integration that provide deeper insights into the biological processes of ADSC reprogramming. Third, long-term safety studies (e.g., NCT03308565) must be finished, followed by efficacy studies in well-characterized patient populations to determine clinical significance and therapeutic benefit [151].

New technologies have particular potential for surmounting existing limitations. CRISPR-based gene editing could enhance neural differentiation efficiency, and 3D bioprinting may improve graft survival and integration [152,153]. Functionalized biomaterial scaffolds (e.g., bioactive matrix-coated nerve conduits) with neurotrophic factors (e.g., pro-survival small molecules) may provide permissive microenvironments for transplanted cells [154,155].

Effective translation of ADSC-derived therapies will require academic–industry collaboration to develop standardized protocols, establish safety and efficacy, and scale up manufacturing processes. Through methodical resolution of current challenges, clinical translation appears attainable for certain neurological indications.

## 7. Conclusions

The induction of ADSCs into iNSCs is a promising neural regenerative therapeutic strategy with the additional benefit of an available autologous cell source and inherent neural regenerative capacity. Induction protocols are currently efficient through the specific modulation of essential signaling pathways, including Notch, Wnt/b-catenin, and Akt/mTOR. Preclinical investigations have also illustrated their ability to promote functional recovery in animal models. Nevertheless, significant hurdles remain, notably in the standardization of protocols, large-scale production, and stringent safety verification. New technique innovations like scRNA-seq, CRISPR-mediated engineering, 3D-bioprinted neural scaffolds, and AI-supported quality control systems are surmounting these hurdles and limitations, and accelerating translational advancement.

ADSC-iNSC-based therapies are expected to advance to clinical trials in the near future. Not only do these therapies show promise for the replacement of dopaminergic neurons, but also for delivering neuroprotection. ADSC-iNSCs thus represent one illustration of convergence between regenerative medicine and precision neurology as a therapeutic strategy for neurodegenerative disease.

## Data Availability

Data sharing is not applicable to this article.

## Abbreviations

AD	Alzheimer’s disease
ADSCs	adipose-derived stem cells
ASCs	adult stem cells
BDNF	brain-derived neurotrophic factor
BME	β-mercaptoethanol
bFGF	basic fibroblastic growth factor
C1	CultureOne
Ca2+	calcium
CGRP	calcitonin gene-related peptide
CNS	central nervous system
CNTF	ciliary neurotrophic factor
CM	conditioned medium
EGF	epidermal growth factor
ESCs	embryonic stem cells
EVs	extracellular vesicles
FGF-2	fibroblast growth factor 2
GDNF	glial-derived neurotrophic factor
hADSCs	human ADSCs
HGF	hepatic growth factor
HPL	human platelet lysate
hPSCs	human pluripotent stem cells
IBMX	3-Isobutyl-1-methylxanthine
iNSCs	induced neural stem cell-like cells
iPSCs	induced pluripotent stem cells
LC	L-carnitine
mRNA	messenger RNA
MSCs	mesenchymal stem cells
NGF	nerve growth factor
NICD	Notch intracellular domain
NSCs	neural stem cells
NT-3	neurotrophin-3
NTN	neurturin
OCD	obsessive-compulsive disorder
PBM	photobiomodulation
PD	Parkinson’s disease
PDGF	platelet-derived growth factor
PKA	protein kinase A
qPCR	quantitative polymerase chain reaction
RA	retinoic acid
RARs	RA receptors
RNA	ribonucleic acid
SCI	spinal cord injury
scRNA-seq	single-cell RNA sequencing
SSRIs	selective serotonin reuptake inhibitors
SVF	stromal vascular fraction
SVZ	subventricular zone
TGF-β	transforming growth factor beta
VEGF	vascular endothelial growth factor
VPA	Valproic Acid
Wnt	Wingless-related integration site
